# Effects of acupuncture versus moxibustion on functional dyspepsia: a randomized clinical trial

**DOI:** 10.1186/s13020-025-01187-x

**Published:** 2025-08-22

**Authors:** Yangke Mao, Pan Zhang, Zhaoxuan He, Yuke Teng, Zilei Tian, Sha Yang, Kuan Fang, Wei Zhang, Yuting Wang, Tao Yin, Fang Zeng

**Affiliations:** 1 Acupuncture and Tuina School, Chengdu University of Traditional Chinese Medicine, Chengdu, 610075 China; 2 Acupuncture and Brain Science Research Center, Chengdu University of Traditional Chinese Medicine, Chengdu, 610075 China; 3 Key Laboratory of Acupuncture for Senile Disease (Chengdu University of TCM), Ministry of Education, Chengdu, 610075 China; 4 Nervous System Disease Treatment Center, Traditional Chinese Medicine Hospital of Meishan, Meishan, 620032 Sichuan China; 5 Department of Hospital Health Management/Physical Examination, Chengdu Fifth People’s Hospital, Chengdu University of Traditional Chinese Medicine, Chengdu, 611130 China

**Keywords:** Functional dyspepsia, Acupuncture, Moxibustion, Complementary and alternative medicine, Epigastric pain, Post-prandial fullness

## Abstract

**Background:**

Functional dyspepsia (FD) is a prevalent gastrointestinal disorder, despite its high prevalence and impact on quality of life, effective treatments are limited. Acupuncture and moxibustion, two complementary therapies based on traditional Chinese medicine, have shown potential in alleviating FD symptoms. However, the differences of acupuncture and moxibustion in FD are unclear.

**Methods:**

A total of 144 eligible FD patients were enrolled and randomly assigned to either the acupuncture or moxibustion group to receive 20 treatment sessions. The primary outcome was the Short-Form Leeds Dyspepsia Questionnaire (SFLDQ) total score after 4 weeks of treatment. Secondary outcomes included SFLDQ symptom-specific score, Nepean Dyspepsia Life Quality Index etc. Linear mixed-effects model was used for analyses.

**Results:**

There was no difference in SFLDQ total score after treatment with acupuncture compared with moxibustion (difference, 0.08[95% CI −0.634 to 0.794], *p* = 0.82), despite both groups were effective. However, the results of the secondary outcomes showed that compared with moxibustion, acupuncture was more effective in alleviating epigastric pain (difference, -0.318[95% CI −0.056 to −0.579], *p* = 0.017) and anxiety mood (difference, −2.893[95% CI −0.419 to −5.367], *p* = .022). On the other hand, moxibustion was more effective than acupuncture in reducing post-prandial fullness (difference, −0.3[95% CI −0.551 to −0.048], *p* = .02). The incidence of adverse events was similar between the groups.

**Conclusions:**

Both the acupuncture and moxibustion groups showed significant improvement in FD symptoms. Although there were no significant differences between the groups at week 4 for the primary outcome, acupuncture exhibited greater improvement in addressing epigastric pain and reduction in anxiety symptoms while moxibustion demonstrated a larger reduction in improving post-prandial fullness. Choice of acupuncture and moxibustion should be tailored to the primary symptoms of FD patients to achieve optimal efficacy.

*Trial registration*: Chinese Clinical Trial Registry (ID: ChiCTR2100049496).

**Graphical Abstract:**

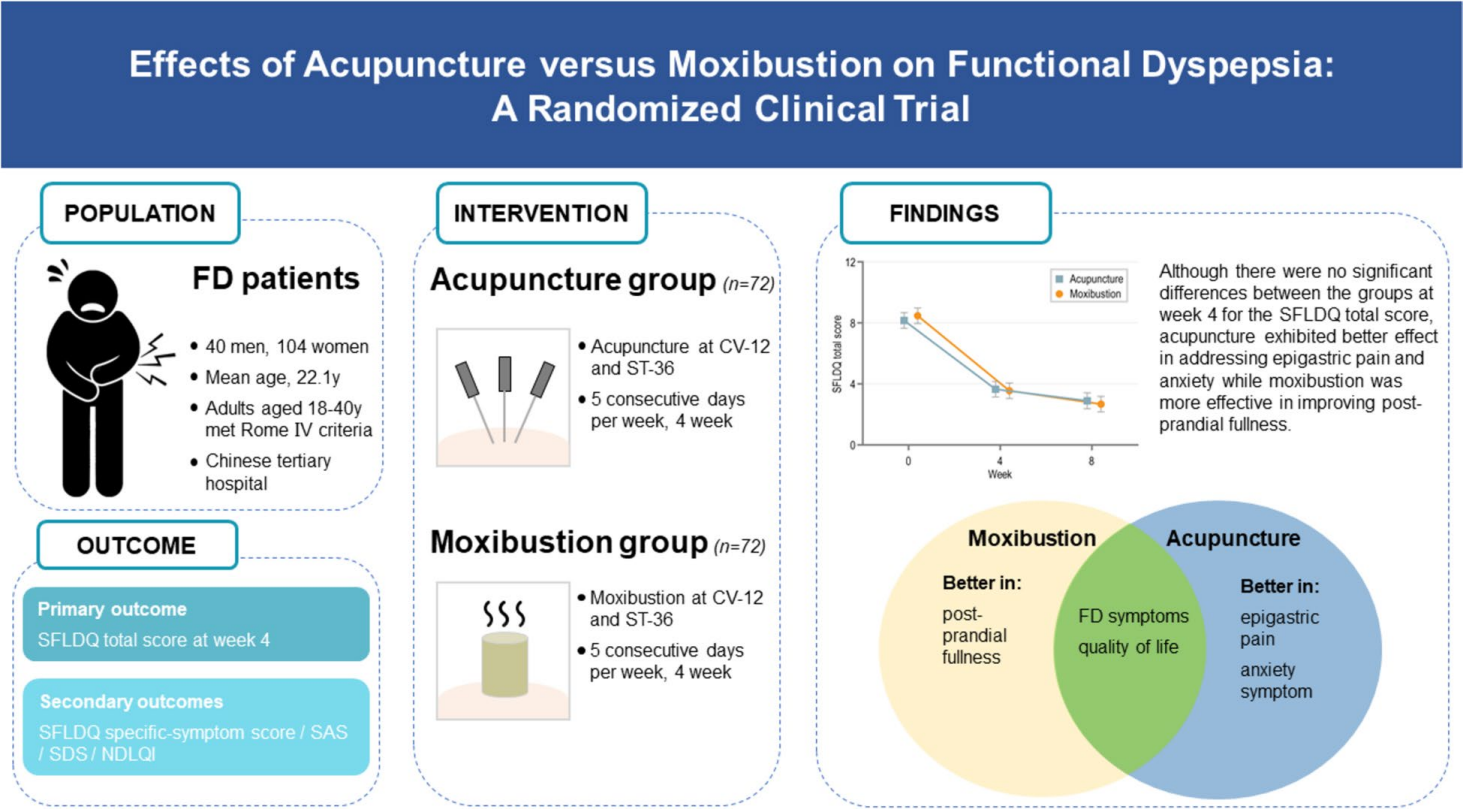

**Supplementary Information:**

The online version contains supplementary material available at 10.1186/s13020-025-01187-x.

## Introduction

Functional dyspepsia (FD), one of the most common functional gastrointestinal disorders (FGIDs), encompasses a constellation of recurrent symptoms, including post-prandial fullness, early satiation, epigastric pain and burning, which remain unexplained by routine medical examinations for organic lesions [1]. As a prevalent worldwide health issue, FD affects approximately 10–16% of global individuals [2], exerts a substantial toll on health and quality of life of patients, resulting in heavy socio-economic burdens, and increases risks of mental disorders such as anxiety [3]. Currently, recommended interventions of FD primarily consist of pharmaceutical approaches, including proton pump inhibitors, prokinetics, etc. However, due to the complex pathophysiology and the drawbacks associated with prolonged medication use, such as high relapse rates and adverse effects [4], complementary and alternative medicine (CAM) therapies are increasingly recognized as promising treatment [5]. A systematic review [6] revealed that the utilization of CAM therapies for FD ranges from 23.6 to 44% in America. In community and university settings, the percentage reaches up to 44–50% [7].

Acupuncture and moxibustion are one of the most widely used CAM therapies and recognized as effective alternative approaches [8]. Several clinical trials have identified their effectiveness and safety in treating gastrointestinal diseases [9, 10]. A systematic evaluation also suggested that these two therapies are more effective in improving quality of life when compared to itopride, domperidone, and sham acupuncture [11]. However, although a preliminary study [12] showed that both acupuncture and moxibustion were significantly effective in treating FD, and some differences were tentatively identified. Nevertheless, the effect difference, symptom-specific benefits of these therapies remain poorly characterized and further research is required. The evidence for how to the two therapies in clinical practice is currently unclear, hindering the advancement of clinical efficacy in FD treatment.

Therefore, we conducted this randomized clinical trial to compare the effects of acupuncture and moxibustion on FD and to further elucidate the respective advantages in addressing the specific symptoms associated with FD. With the result of enhancing our understanding of the therapeutic benefits of acupuncture and moxibustion, clinicians may consider acupuncture or moxibustion based on the main symptoms of FD patients to maximize the treatment benefits.

### Study design

This randomized clinical trial was conducted at Hospital of Chengdu University of TCM from September 2021 to July 2023. The study consisted of two periods: a 4-week treatment phase followed by 4-week post-treatment follow-up period. The study protocol was approved by Sichuan Regional Ethics Review Committee on Traditional Chinese Medicine (ID: 2021KL-059). The trial was carried out in accordance with the principles of the Declaration of Helsinki. Additionally, the protocol was registered with Chinese Clinical Trial Registry (ChiCTR2100049496). This study followed the Consolidated Standards of Reporting Trials (CONSORT) guideline and the Standards for Reporting Interventions in Clinical Trials of Acupuncture (STRICTA) guideline.

### Participants

The recruitment strategy primarily comprised advertisements and free medical consultations. Participants who met all the following criteria were included: (1) the Rome IV criteria for FD, (2) between 18 and 40 years old, (3) had not taken any gastrointestinal-related treatments for at least 2 weeks prior to enrollment, and (4) had not taken part in any clinical trials within the previous 3 months. Prior to group allocation, comprehensive assessments, including physical examination, routine laboratory tests, carbon-14 urea breath test, electrocardiogram, abdominal ultrasonography, and upper gastrointestinal endoscopy, were conducted to exclude participants with potential organic lesions. We also excluded participants if they had: (1) any serious disease that could cause dyspeptic symptoms; or (2) organic or other functional gastrointestinal diseases; or (3) a history of gastrointestinal surgery; or (4) pregnancy or lactation; or (5) contraindications to acupuncture or moxibustion, such as needle sickness, skin damage at the acupoint sites, allergy to moxa, or infections. All included participants gave written informed consent.

### Randomization and blinding

The included patients were randomly assigned in a 1:1 ratio to either the acupuncture or moxibustion group. The random sequence was generated by an independent mathematician, who was not involved in the study, with PASS software 15.0 (NCSS, LLC., Kaysville, U.T., USA). Due to the nature of the two interventions, blinding of patients and acupuncturist was not feasible. However, patients were organized in different treatment rooms and each patient was separated in a closed unit. The acupuncturist was not involved in data collection or data analysis, and outcome assessor, data managers, and statisticians were blinded throughout the entire study.

### Intervention

All patients in both groups received 20 sessions of treatment over the course of 4 weeks (5 consecutive days per week) and the same acupoints *Zhongwan* (CV-12) and *Zusanli* (ST-36), which are the most commonly used acupoints for FD and have been proven to be effective in previous studies [13]. One licensed acupuncturist, with at least 3 years of clinical experience and certified through a 2-day standard training course, administered all the interventions. The published protocol [14] and Supplementary 1 presented more detailed information on the acupuncture and moxibustion interventions.

### Outcomes measures

All clinical assessments were conducted at three time points: week 0 (baseline), week 4 (after treatment) and week 8 (follow-up). The primary outcome measure was the Short-Form Leeds Dyspepsia Questionnaire (SFLDQ) total score at week 4. The SFLDQ encompasses six major dimensions of symptoms: epigastric pain, post-prandial fullness, early satiety, epigastric burning, nausea and belching. A five-point Likert scale ranging from absent (0 point) to very severe (5 points) was used to grade the severity of the symptoms, with higher scores indicating more severe dyspepsia symptoms.

The secondary outcome measures included the SFLDQ symptom-specific score, and the Nepean Dyspepsia Life Quality Index (NDLQI) score. The SFLDQ symptom-specificity score contained various dimensions of dyspeptic symptoms, such as epigastric pain, post-prandial fullness and others. This provided a more detailed assessment of each individual symptom, allowing for a better understanding of how each treatment affects specific symptoms of FD. The NDLQI was utilized to assess FD patients' specific quality of life experienced by FD patients in relation to their symptoms. Low scores meant poor quality of life. Besides, the Zung Self-rating Anxiety Scale (SAS) and the Zung Self-rating Depression Scale (SDS) were also collected to evaluate mental health status. Adverse events potentially related to acupuncture or moxibustion were recorded over the trial period, such as subcutaneous hemorrhage, residual pain, scald and infection.

### Statistical analysis

The minimal sample size of 124 (62 in each group) was determined based on the findings of a pilot study [12] that focused on the changes in the SFLDQ in response to a 4-week acupuncture intervention versus moxibustion intervention. The study revealed a mean improvement of 5.62 in the SFLDQ total score for the acupuncture group and 3.56 in the moxibustion group. To calculate the sample size, a standard deviation of 3.5 was chosen with 90% statistical power and a two-tailed alpha level of 0.05, in accordance with the reference and our clinical experience. Finally, to account for an estimated dropout rate of 15%, a total of 144 participants was planned to be recruited.

All data analyses were conducted with R (version 4.4.2) based on the intention-to-treat principle of all randomly assigned patients. The descriptive analysis was used for the baseline characteristics of the patients in each group. The primary outcome and all secondary outcomes were assessed by linear mixed-effects model. A *p* < 0.05 was considered statistically significant and all statistical tests were two-sided.

## Results

### Baseline characteristics

**Fig. 1:**
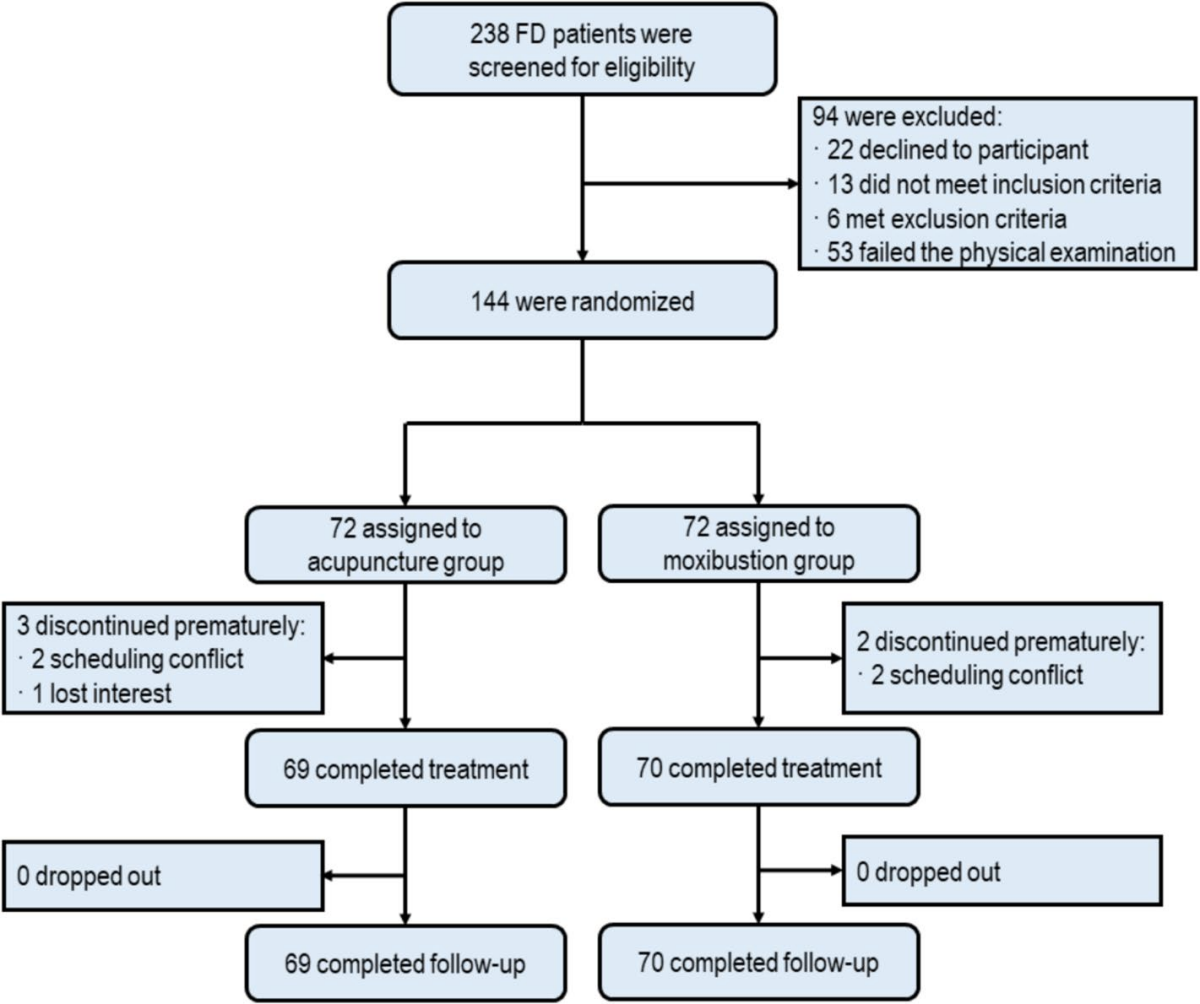
The study flowchart

A total of 238 FD patients were initially screened. Of these, 144 eligible patients were enrolled and randomly assigned to either the acupuncture or moxibustion group, including 104 women (72.2%) and 40 men (27.8%) with a mean (SD) age of 22.1 (0.1) years. A total of 139 patients completed the entire trial, while 5 patients withdrew from the trial before receiving first treatment owing to personal issues, with 3 in the acupuncture group and 2 in the moxibustion group (Fig. 1). The baseline demographic and clinical characteristics of the patients were balanced between groups (Table 1).

**Table 1:** Baseline demographic and clinical characteristics of the intention-to-treat population

Characteristic	Study group^a^	
	Acupuncture (n = 72)	Moxibustion (n = 72)
Age, y	22.35 (0.23)	21.88 (0.20)
Duration of FD, mo	39.42 (3.67)	37.39 (2.86)
Sex, %		
Men	19 (26.3%)	21 (29.1%)
Women	53 (73.6%)	51 (70.8%)
BMI, kg/m^2^	20.68 (0.31)	20.44 (0.28)
Educational attainment, y	15.07 (0.23)	14.64 (0.18)
SFLDQ		
Total score	8.15 (3.03)	8.38 (2.41)
Epigastric pain	1.51 (1.16)	1.52 (1.05)
Post-prandial fullness	2.03 (1.12)	2.14 (1.10)
Early satiety	1.47 (0.11)	1.49 (0.10)
Epigastric burning	0.83 (0.12)	0.90 (0.11)
Nausea	0.92 (0.10)	1.10 (0.12)
Belching	1.39 (0.12)	1.51 (0.11)
NDLQI	77.28 (1.17)	79.62 (0.90)
SAS	43.85 (1.09)	43.73 (1.13)
SDS	45.26 (1.23)	45.55 (1.28)

*BMI* Body Mass Index, *NDLQI* Nepean Dyspepsia Life Quality Index, *SFLDQ* Short-Form Leeds Dyspepsia Questionnaire, *SAS* Zung Self-rating Anxiety Scale, *SDS* Zung Self-rating Depression Scale

^a^Unless otherwise stated, data are presented as mean (SD)

### Primary outcome

**Fig. 2:**
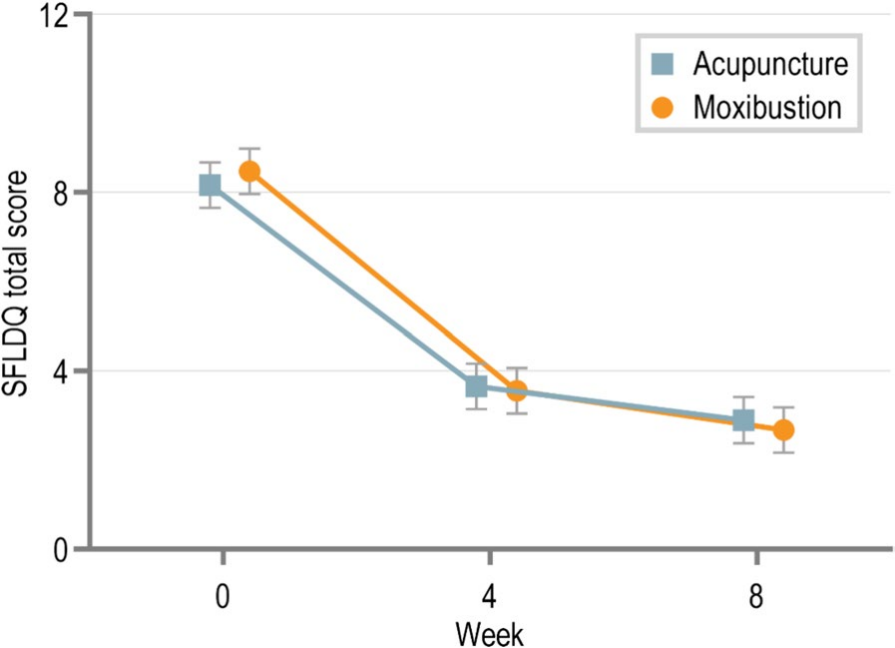
Changes over time in the SFLDQ total score

For the primary outcome measure, the SFLDQ total score at week 4 demonstrated significant improvement with 3.663 (95%CI, 3.156 to 4.169) in the acupuncture group, and 3.742 (95% CI 3.239–4.246) in the moxibustion group (Table 2). However, there was no statistically significant between-group difference (difference, 0.08 [95% CI −0.634 to 0.794],* p* = 0.826). The trends of SFLDQ total score from baseline to follow-up in the 2 groups are shown in Fig. 2.

**Table 2:** Comparison of the primary outcome in the acupuncture group and moxibustion group

Outcome	Mean score (95% CI)		Moxibustion vs Acupuncture	
	Acupuncture group	Moxibustion group	Difference (95%CI)	*p* value
SFLDQ total score				
Week 4	3.663 (3.156–4.169)	3.742 (3.239–4.246)	0.08 (−0.634 to 0.794)	0.826
Week 8	3.184 (2.678–3.691)	3.071 (2.568–3.574)	−0.113 (−0.827 to 0.601)	0.755

*SFLDQ* Short-Form Leeds Dyspepsia Questionnaire

### Secondary outcomes

**Fig. 3:**
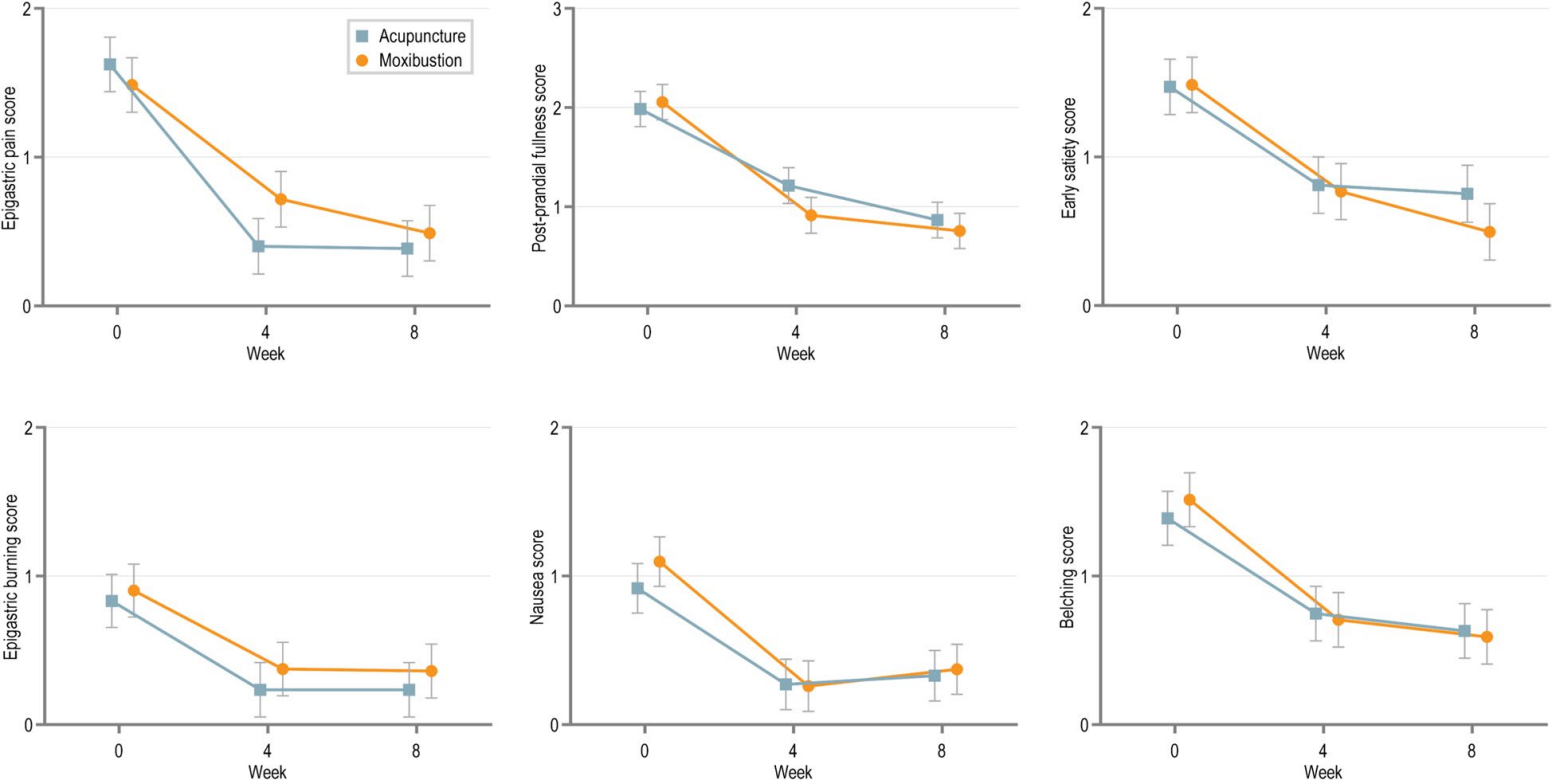
Changes over time in the SFLDQ symptom-specific score

**Fig. 4:**
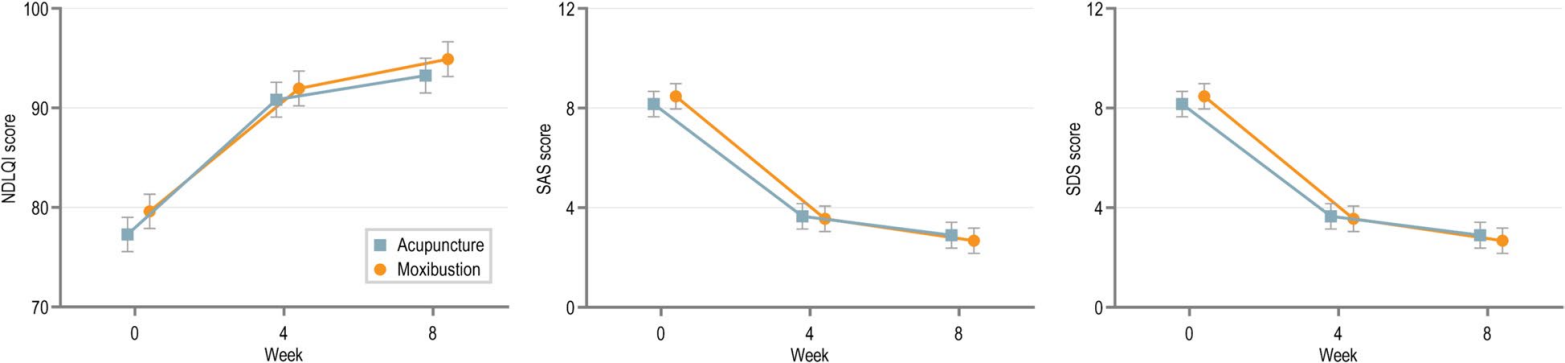
Changes over time in the NDLQI, SAS and SDS

The results for each of the secondary outcomes are presented in Table 3. Although no significant between-group difference was identified in the SFLDQ total score at week 4, significant differences were found in FD symptom-specific scores and anxiety symptoms. The decrease in the epigastric pain score (0.318, [95% CI 0.056–0.579], *p* = 0.017) and SAS (2.893, [95% CI 0.419–5.367], *p* = 0.022) of the acupuncture group was significantly better than the moxibustion group at week 4. The moxibustion significantly reduced post-prandial fullness score compared with the acupuncture group (-0.3, [95% CI −0.551 to −0.048], *p* = 0.02). Anyway, similar to the primary outcome, the remaining secondary outcomes achieved generally similar improvement but did not attain statistical significance, including early satiety, epigastric burning, nausea, belching scores, NDLQI and SDS (Figs. 3 and 4).

**Table 3:** Comparison of the secondary outcomes in the acupuncture group and moxibustion group

Outcomes	Mean score (95% CI)		Moxibustion vs Acupuncture	
	Acupuncture group	Moxibustion group	Difference (95%CI)	*p* value
SFLDQ symptom-specific score				
Epigastric pain				
Week 4	0.4 (0.214–0.585)	0.718 (0.533–0.902)	0.318 (0.056 to 0.579)	0.017*
Week 8	0.385 (0.2–0.571)	0.489 (0.305–0.673)	0.104 (−0.158 to 0.365)	0.436
Post-prandial fullness				
Week 4	1.213 (1.035–1.392)	0.914 (0.737–1.091)	−0.3 (−0.551 to −0.048)	0.020*
Week 8	0.866 (0.687–1.044)	0.757 (0.579–0.934)	−0.109 (−0.36 to 0.143)	0.395
Early satiety				
Week 4	0.81 (0.621–0.999)	0.767 (0.58–0.955)	−0.043 (−0.309 to 0.224)	0.753
Week 8	0.752 (0.563–0.941)	0.496 (0.308–0.683)	−0.256 (−0.522 to 0.01)	0.059
Epigastric burning				
Week 4	0.235 (0.055–0.415)	0.375 (0.196–0.554)	0.14 (−0.114 to 0.394)	0.279
Week 8	0.235 (0.055–0.415)	0.361 (0.182–0.54)	0.126 (−0.128 to 0.38)	0.331
Nausea				
Week 4	0.271 (0.104–0.439)	0.259 (0.093–0.425)	−0.012 (−0.249 to 0.224)	0.918
Week 8	0.329 (0.162–0.497)	0.373 (0.207–0.54)	0.044 (−0.192 to 0.28)	0.714
Belching				
Week 4	0.747 (0.566–0.928)	0.706 (0.525–0.886)	−0.041 (−0.297 to 0.214)	0.750
Week 8	0.631 (0.45–0.812)	0.591 (0.411–0.771)	−0.04 (−0.295 to 0.216)	0.760
NDLQI				
Week 4	90.84 (89.104–92.577)	91.96 (90.234–93.685)	1.12 (−1.328 to 3.568)	0.369
Week 8	93.261 (91.525–94.997)	94.923 (93.197–96.649)	1.662 (−0.786 to 4.11)	0.183
SAS				
Week 4	31.487 (29.733–33.242)	34.38 (32.636–36.124)	2.893 (0.419 to 5.367)	0.022*
Week 8	30.817 (29.062–32.571)	31.276 (29.532–33.02)	0.46 (−2.014 to 2.934)	0.715
SDS				
Week 4	34.891 (32.807–36.976)	34.774 (32.7 to 36.847)	−0.118 (−3.058 to 2.822)	0.937
Week 8	32.149 (30.065–34.234)	32.631 (30.557–34.704)	0.482 (−2.459 to 3.422)	0.747

*NDLQI* Nepean Dyspepsia Life Quality Index, *SFLDQ* Short-Form Leeds Dyspepsia Questionnaire, *SAS* Zung Self-rating Anxiety Scale, *SDS* Zung Self-rating Depression Scale

### Adverse events

During the 4-week treatment period, five participants experienced treatment-related adverse events, with 3 cases (4.1%) in the acupuncture group (one patient reported persistent tingling sensation after treatment, the other two exhibited subcutaneous hemorrhage) and 2 (2.7%) in the moxibustion group (one patient described feelings of nausea during treatment, while another one reported instance of scald and itching). The total rate of adverse events was 3.4%, and the rate of adverse events was not significantly different between the two groups (*χ*^2^ = 0.223, *p* = 0.637). All adverse events were of mild severity and deemed tolerable, and all affected participants were provided with routine care. No participants withdrew from the trial due to adverse events.

## Discussion

This randomized clinical trial demonstrated that acupuncture and moxibustion are both effective for treating FD, although no significant between-group differences were observed in the primary outcomes. Exploratory analyses of secondary outcomes suggested that acupuncture was associated with greater reductions in epigastric pain and anxiety symptoms, while moxibustion showed larger improvements in post-prandial fullness. Compare with the rate of adverse event of medications recommended in FD (ranges from 11.6 to 35.6%) [15], the rate in acupuncture and moxibustion group was 4.1% and 2.7% respectively, with no severe adverse events reported during the trial, suggesting that acupuncture and moxibustion are effective and safe treatments for FD patients.

In our study, the clinically significant improvement in dyspeptic symptoms, quality of life and mental health status were observed in the acupuncture and moxibustion group, and therapeutic effects after treatment were maintained for a period of least 4 weeks. At the end of 4-week treatment, although there was no statistically significant difference in SFLDQ total score between the acupuncture and moxibustion group (difference, 0.08, *p* = 0.83), there were effect differences with statistically significant differences in FD specific-symptoms and emotional symptoms. In the present study, moxibustion was found to be more effective in dyspeptic symptom of post-prandial fullness than acupuncture, and acupuncture was better than moxibustion on alleviating epigastric pain and reducing FD patients with anxiety status.

Acupuncture and moxibustion, originating in ancient China, have been used to treat gastrointestinal diseases in China for thousands of years. Previous studies have indicated that both treatments were effective on treating FGIDs [16, 17] and inflammatory bowel disease [18]. However, although both treatments based on acupoints, as two different traditional Chinese medicine treatments, the differences in their respective effects remain unclear [19], and there have been no similar randomized controlled clinical trials focusing on the effects of acupuncture versus moxibustion on treating FD. Therefore, we strictly conducted this trial to provide clinical evidence that acupuncture and moxibustion have different therapeutic effects on FD symptoms and mental health status, and to help maximize the benefits of these treatments on FD based on the predominant symptom.

Although acupuncture and moxibustion mechanisms are not well understood, previous studies may provide some plausible explanations why acupuncture and moxibustion has different effects on FD. As a typical disorder of gut-brain interactions [20], FD was found to commonly exhibit spontaneous functional activity abnormalities in "pain matrix" areas such as the insula and anterior cingulate gyrus, and these brain regions were also involved in emotional regulation [21, 22]. Notably, after acupuncture treatment, there was a corresponding improvement in the activity of these pain/emotion-associated brain regions, including resting-state activity [23] and functional connectivity [24], and were significantly correlated with clinical symptom improvement [25]. Interestingly, multiple studies have also revealed that acupuncture has significant effects on treating visceral pain [26, 27], and that efficacy related to regulation of brain functions [28, 29]. Therefore, we can infer that the more remarkable modulation on abnormal brain regions including the insula and anterior cingulate gyrus might be the specific mechanism of acupuncture. Compared to acupuncture, several studies indicated that moxibustion may mediate patients with gastrointestinal function disorder in dysregulated default mode network regions [30, 31], which closely contribute to gastrointestinal motility disturbances and aberrant central processing of visceral sensation [32]. Besides, some animal studies [33, 34] showed that moxibustion may improve gastrointestinal motility by regulating the stability and abundance of the intestinal microbiota. Other evidence [35, 36] indicated that moxibustion significantly ameliorates abnormal gastric electrophysiological parameters in FD patients, as evidenced by marked elevations in gastric slow wave frequency and motility index. Thus, a plausible hypothesis suggested that the sustained, comfortable warm stimulation of moxibustion generated positive feelings of gratification and warmth, and may enhance gastric accommodation and accelerate emptying [37]. Additionally, incomplete blinding may have differentially modulated placebo effects. Moxibustion's heat sensation may amplify gut-focused placebo responses via visceral-somatic interaction [38], while acupuncture's stronger somatosensory input might enhance expectancy effects for neurological symptoms, such as the reduction in epigastric pain and anxiety [39].

It is important to note that similar comparative studies examining the effects of acupuncture and moxibustion for other diseases have also been conducted, providing additional context for the feasibility and necessity of this trial. Similarly, trials on irritable bowel syndrome revealed analogous symptom-specificity: acupuncture demonstrated better reduction for anxiety and depression emotion, while moxibustion reported greater improvements in gastrointestinal motility especially defecation-related symptoms [40, 41]. Research by Bao et al. [30] on Crohn's disease found both therapies beneficial but with differing central mechanisms in modulating brain homeostatic afferent processing network and default mode network, respectively. Another study [42] on chronic fatigue syndrome showed differences in terms of the duration of therapeutic effects, with acupuncture having a significant immediate effect and moxibustion showing beneficial long-term effects. These cross-disease trials confirmed that the potential therapeutic symptom-specificity of acupuncture and moxibustion, and supported for the necessary of comparative evaluation of these therapies in FD.

This study has several limitations. First, due to the characteristics of acupuncture and moxibustion, it was not possible to blind the acupuncturist and patients during the treatment sessions, which might introduce bias. Second, although a higher proportion of female FD patients were included in this study because of the higher incidence of FD in females [43], there was no significant gender difference between groups. However, it may limit the generalizability of the findings to the male population. Future studies should aim for a more balanced gender distribution to ensure broader applicability. Third, due to most patients had the overlap in symptoms between postprandial distress syndrome and epigastric pain syndrome subtypes, we did not differentiate between them in this study. However, the respective advantages of the two treatments in this study suggest that future research should focus on the efficacy of the two treatments for FD subtypes. Forth, the interpretation of symptom-specific differences may be limited by the modest effect sizes and limited power. Finally, the follow-up period of 4 weeks may not fully capture the long-term effects of acupuncture and moxibustion on FD symptoms. Future studies with longer follow-up durations are needed.

## Conclusions

In conclusion, there are effect differences between acupuncture and moxibustion, and that each has respective therapeutic advantages on FD. Moxibustion was more effective in improving post-prandial fullness, while acupuncture showed better benefits in alleviating pain-related symptoms and anxiety symptom. These results provide evidence for clinicians to reasonably adopt more suitable treatment approaches according to predominant FD symptoms.

## Supplementary Information

Supplementary material 1.

## Data Availability

Reasonable requests for the original data can be sent to the corresponding author.

## Abbreviations

CAM: Complementary and alternative medicine
CONSORT: Consolidated standards of reporting trials
FD: Functional dyspepsia
FGIDs: Functional gastrointestinal disorders
NDLQI: Nepean dyspepsia life quality index
SAS: Zung self-rating anxiety scale
SDS: Zung self-rating depression scale
SFLDQ: Short-Form Leeds Dyspepsia Questionnaire
STRICTA: Standards for Reporting Interventions in Clinical Trials of Acupuncture

## Declarations

### Clinical trial registration

The trial was carried out in accordance with the principles of the Declaration of Helsinki. Additionally, and was registered with Chinese Clinical Trial Registry (ChiCTR2100049496).
