# Supplementary material for: Effects of acupuncture versus moxibustion on functional dyspepsia: a randomized clinical trial
Source: Chin Med. 2025 Aug 22;20:131. doi: 10.1186/s13020-025-01187-x (PMC12372385; doi:10.1186/s13020-025-01187-x)
Supplement: Supplementary file 1 — Supplementary material 1. [file 13020_2025_1187_MOESM1_ESM.docx]

**Supplementary materials**

**Supplementary 1. Details on materials, prescriptions and treatment manipulation of acupuncture and moxibustion**

Acupuncture needle is a sterile, disposable and filiform needle with length of 40 mm and diameter of 25 mm (*Huatuo* Medical Instrument Co., Ltd., China). Moxibustion is a portable, disposable, and pure moxa stick with length of 60 mm, diameter of 41 mm and 13±1g weight (*Bozhou Aikeshu* Medical Co., Ltd., China) which could be securely placed on acupoint its own adhesive tape.

FD patients received 20 sessions acupuncture or moxibustion treatment in 4 weeks (5 consecutive days per week, then 2 days off). *Zhongwan* (CV-12) and *Zusanli* (ST-36) were selected as the treatment prescription (**Figure S1**). Acupuncturist alternatively used unilateral *Zusanli* (ST-36) acupoints (left and right) in each session. All treatment manipulation was conducted by one licensed acupuncturist with clinical experience over 3 years who had our certified 2-day standard training course (the details of the course are as follows: 1) a two-day, 12-hour training course provided by senior doctors; 2) standardized acupuncture techniques (e.g. how to insert the acupuncture, neutral reinforcing-reducing needle manipulation technique with uniform lifting-thrusting and twirling); 3) regulated communication phrases for dialogue.; 4) how to handle adverse events (e.g. subcutaneous haemorrhage, post-needling localized pain) and etc.).

Acupuncture treatment session was as follows: After skin sterilization, the needles were perpendicularly inserted 25 to 30 mm into acupoints. Then the needles were evenly twisted between 90° and 180°, lifted and thrusted with the amplitude of 3-5 mm, 60 to 90 times per minute to achieve *Deqi* sensation (a mixed sensation includes soreness, distention, numbness which indicates clinical efficacy). Once patient felt *Deqi* sensation, needles were retained in the acupoints for 30 minutes with the aforementioned procedure manipulated for 10-15s every 10 minute in order to maintain the *Deqi* sensation.

Moxibustion treatment session was as follows: The moxa sticks were ignited and then attached to acupoints with its own adhesive tape for 30 minutes. During the 30 minutes, the temperature of the portable moxibustion would be adjusted according to patient's feelings and tolerance, in order to make patient feel warm rather than burning pain and prevent skin scald.

| 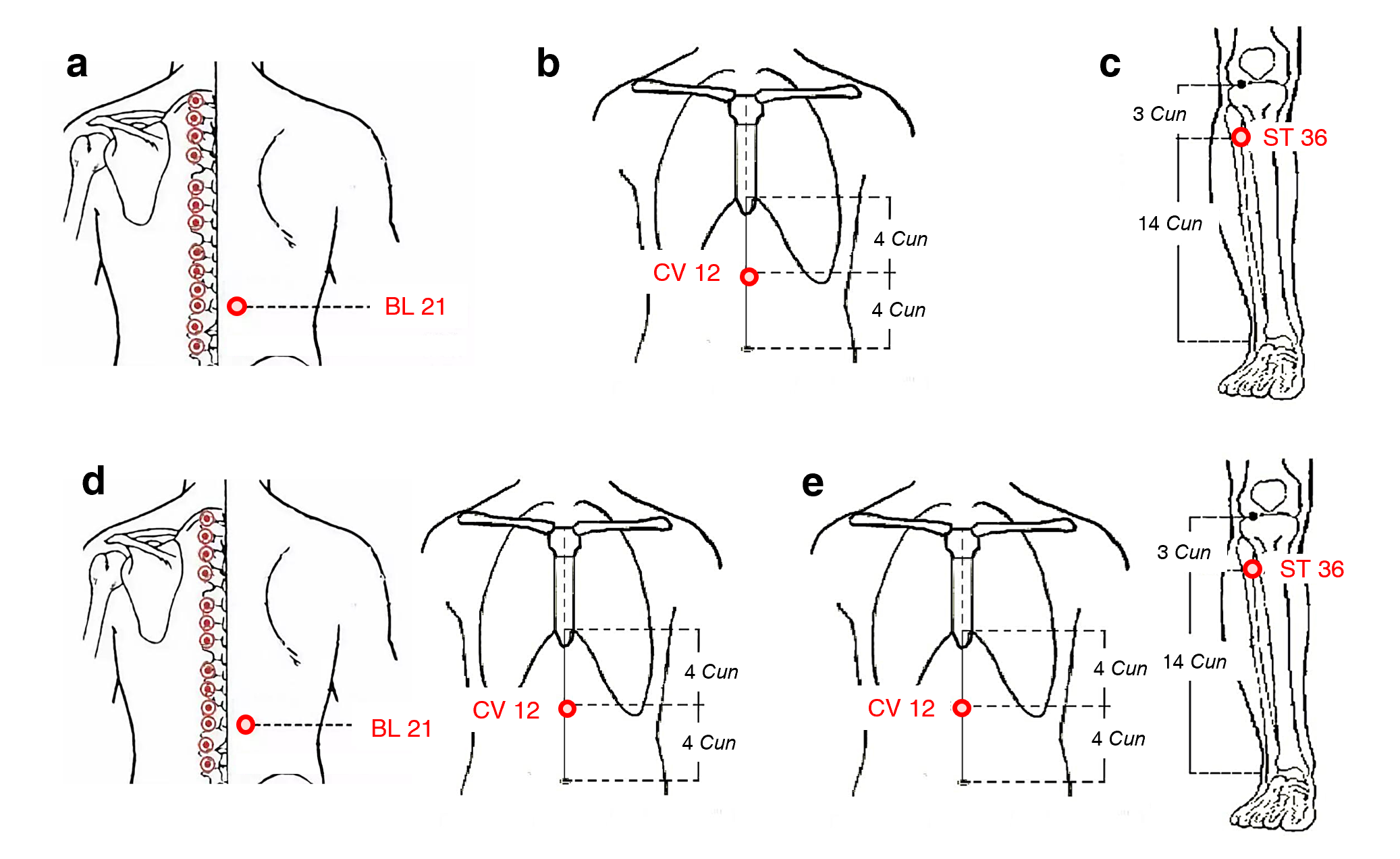 |
| --- |
| **Figure S1** \| The location of acupoints |
